# Seventh Annual Session of the Maryland State Dental Association

**Published:** 1890-10

**Authors:** 


### SEVENTH ANNUAL SESSION OF THE MARYLAND STATE DENTAL ASSOCIATION.

(Continued from page 551.)

### REPORT OF COMMITTEE ON DENTAL LITERATURE.

It is not to be expected that a youthful and vigorous profession like ours, which touches and fraternizes in the most friendly way with medical science and mechanical arts, which rubs elbows with business, and hobnobs with commercial enterprise, should have in its brief existence accumulated a large and extended literature. Dentistry is just now vigorously aggressive and pushing forward, rather than employed in recording what it has done, and has had but little time for contemplation or retrospection. The reports from the various dental associations smack of conflict and tell of an active, throbbing life, vigorous, energetic, and inspiriting; in fact, we may justly say of dentistry that it is too busy making history to have time to write it.

Nevertheless, much may be said of the records of our achievements, for that about comprises our literature. The literature of our profession is keeping pace with its development, and, in the main, is a faithful record of its methods and results. The close of the year marks the production of numerous text-books in dentistry, the mere enumeration of which were an unprofitable task, and the discussion of which, neither my time nor your patience will allow. It is not possible, however, to refrain from recording the feeling of gratification in noting that in German, French, and English have appeared technical works, whose object is to treat as a whole the system of dentistry. The study of the profession as a distinct calling is thus seen to be an assured fact, and a worthy aim of scientific men; this, of itself, independent of the value of the works referred to, is a fact upon which the profession should be congratulated.

Among other evidences of this same fact and in recognition of the discriminating continuity of effort as well as of ripe experience and mature judgment, dentists may note with pride that the work of securing in a series of volumes, exhaustive and learned treatises upon all subjects relating to dentistry, began several years ago by Professor Wilbur F. Litch, has been successfully accomplished, and the "American System of Dentistry," we predict, will long be the standard work of reference in this country, and will add lustre to the name of American dentistry abroad.

A resolution introduced into the Association of Faculties in 1886 is beginning to bear fruit in the appearance of text-books, bearing the approval of that association, and evidencing the scrutinizing care, skilful arrangement, and clear and forcible expression of experienced teachers. The completion of this series of text-books will mark a new era in dental education.

Much that has been written in the last year in the journals has been descriptive of bridge-work and the various methods of its preparation; and Dr. Evans has exhibited rare skill and judgment in compiling and selecting from these, furnishing the profession a book upon bridge-work, in which may be found a description of all the best methods.

The journals have performed and are performing their function with skill and ability. They publish with fidelity and without prejudice the proceedings of associations and original communications. There is no reason to complain that we do not get an opportunity to be heard, for in some one of the various journals the ambitious and able dentist can always find an impartial forum from which to address and teach his brethren. It is largely, we think, to be imputed to this spirit of fairness, this desire to exhibit, as they are, the results of professional labor and investigation, that it has sometimes happened that our desire for a fuller editorial column has gone unsatisfied; the editors have wisely considered it best to reproduce the thoughts and opinions of the body of the profession rather than those they individually hold.

We have been glad, then, to note the very marked improvement in the majority of the journals. The best are remarkably clear and free from obnoxious and distasteful advertising puffs, and from an improper appropriation of reading pages to that purpose,—and this despite the fact that most of the journals are published by dental depots and dental-supply firms. In fact, the general dignity and correctness of our publications compare most favorably with those of other professions. If it be not invidious to discriminate, we would like, with no little pride and with the confident hope for its success, to refer to the recent organization of a society of the most prominent men in our profession, under whose direction and with whose support the INTERNATIONAL DENTAL JOURNAL has made its appearance as the successor to the *Independent Practitioner*. As this is an effort at independent journalism, it certainly demands the support and encouragement of every man in the dental profession who has its advancement at heart. We are glad to report that this journal is ably edited, has been successful, and is making new friends every day.

Your committee regret that scantiness of time has made it absolutely impossible to render a report worthy of the profession and of the occasion. They can but note impressions and suggestions which the rapidly enlarging literature of dentistry has made upon them ; can but convey to you a hint of that ever-present sense of activity, development, and growth with which the profession and its literature impress them ; and congratulate you that we are awake to the demands of our chosen calling and moving towards the goal.

B. HOLLY SMITH,
*Chairman.*

DISCUSSION ON REPORT OF COMMITTEE ON PUBLICATION AND DENTAL LITERATURE.

*Dr. F. J. S. Gorgas.*—Mr. President, I would be glad to hear Dr. W. X. Sudduth make some reply in reference to that part of the subject which pertains to the great influence of editors. I desire simply to make one suggestion of a practical nature, which has just occurred to me. I refer to the difficulty of procuring from the members of the dental profession contributions of articles for publication, and I assume that in this respect the experience of publishers of dental journals is the same everywhere. While, as a rule, the members of the medical profession seem disposed to ventilate their views and to give the public the benefit of their experience, we find, on the part of members of the dental profession, what we may call a diffidence to do likewise; and this diffidence seems to exist to a degree corresponding with the disposition of medical men to be heard.

It is indispensable to the success of a journal devoted to a specialty that it should be instructive and attractive; but this it cannot be unless members engaged in following that specialty lend their assistance towards the accomplishment of that end. If our journals were able to secure contributions by paying for them sums of money the case might be different, for the stimulus of compensation would certainly produce valuable results. Certainly every dental journal in the country would be improved if each would maintain on hand a sufficient sum for the encouragement of such talent as is to be found in the ranks of our profession.

The publishing of dental journals is an up-hill labor when the members of the profession do not contribute literary matter by way of support. We have experienced that difficulty here in Baltimore, and perhaps to a greater extent than it has made itself felt in the case of the INTERNATIONAL DENTAL JOURNAL, because a publication like that can undoubtedly command more literary support than can be made available in our own city.

I may say here that we have been much pleased with the recent appearance of the INTERNATIONAL DENTAL JOURNAL. It presents evidences of marked improvement in contrast with its former appearance.

*Dr. W. Xavier Sudduth.*—Mr. President, Dr. Gorgas has spoken upon a point that is well deserving of attention, and that is in regard to the production of literature or the possibilities of obtaining literature for dental journals,—a question to which I have given more thought, perhaps, during the last eighteen months or two years than to any other question.

Dr. Gorgas has advanced a statement upon which I differ with him, and that is that if dental journals were supplied with sufficient money to enable them to pay for literature they would get it. I would say to the doctor that I do not believe they would get it. I do not believe there is money enough in the coffers in any of the manufacturing centres of this country to buy the literature of the profession. It is not for sale. The only way you can get it is by the stimulus of personal appeals before dental societies. Hired writers in this country will not succeed. It is only through personal intercourse and through the pressure that is brought to bear upon members of the profession by committees that we can get literature for dental journals; and if any of you have served on such committees you will probably understand how the pressure is brought to bear.

Success in dental journalism in this country is to be most readily attained by the publisher or editor making himself known to or identified with dental societies. As an editor and publisher of a dental journal I have made it a special rule to make myself known to dental societies; and through them I have sought to secure the literature which was necessary for our journal. I have experienced, as Dr. Gorgas has said he has experienced in connection with his journal, in our work a great deal of difficulty in getting good literature; and it has seemed sometimes as though it were almost impossible to look ahead for six months and foresee sufficient material for publication of an interesting character through that period.

Another consideration is that when the contributions of members who have been appealed to do not appear in the JOURNAL, they are apt to take offence at me as the publisher for that reason. But it should be recollected that the editor must be allowed to exercise some discrimination in the selection of matter which is to appear; otherwise his publication would not be up to the standard. I would like to say to you, therefore, although I am a stranger to many of you, that if at any time you feel that your productions have been slighted, you should remember that the editor is in a very trying position, that it is hard for him to please everybody, and that, above all other considerations, he must have the interest of his journal at heart rather than the interest of individual members of the profession.

*Dr. Kirk.*—Mr. President, I called Dr. Sudduth out in the expectation that he would give us a different side of the story from that which he has stated, but he has not done that, and I will tell it myself.

Dr. Smith has called attention, in his report, to the necessity of supporting our dental journals. Now, the success of a dental journal does not depend upon what is published in it or upon how much matter is accumulated for it, but it rather depends largely on how that journal is supported by the profession at large. I thought that Dr. Sudduth would like to say something upon that phase of the question,—in other words, upon the subscription list. After the cream of the thought of the general profession has been put into printed form it is disheartening at times to observe how little interest is taken in the matter when it is presented to the profession in that shape. There seems to be a feeling of general apathy among the profession on this point. I may say it is humiliating to find how small a proportion of men engaged in dentistry ever read a dental journal. That point is one that I would like to see enlarged upon by discussion. The practical question is how many of the men engaged in dentistry actually subscribe to dental journals and pay their subscriptions?

*Friday, December* 6, 1889.—*Afternoon Session.*

### DISCUSSION ON PUBLICATION AND DENTAL LITERATURE RESUMED.

Dr. R. Findlay Hunt suggested a correction of that part of the minutes of the morning session, as read by the secretary, which purported to give the substance of the resolution which was adopted on motion of Dr. Grady, concerning the preservation of sets of the *Dental Cosmos*, etc. He explained that the resolution, before being voted upon, had been modified, he thought, so as to empower the Committee on Publication to act on the subject according to their discretion.

### AN APPEAL FOR THE DENTAL PROTECTIVE ASSOCIATION.

Dr. R. B. Winder spoke briefly in advocacy of the claims of the Dental Protective Association to recognition and support by the dental profession generally.

Referring to a letter he had received from Dr. Crouse (who, he said, was the head and front of the protective movement), Dr. Winder explained that the organization had been specially designed for the defence of dental operators against encroachments upon their rights by the International Tooth Crown Company. The Protective Association pledged itself to defend the case of any of its members who might be sued by that company. He said that the movement for mutual protection has extended quite generally throughout the country, particularly in the north, where bridge-work is more frequently made use of than elsewhere. He assured his hearers that the individual protection which has been guaranteed is an actual existing fact, as he knew of no instance in which a member of the Protective Association has been interfered with since the formation of that organization. He urged that it was both a necessity and a duty for the profession to strengthen itself by resistance not only in this, but in every other case in which it has been attempted to foist a fraudulent patent upon it.

He denounced the International Tooth Crown Company as having attempted to gain its end by a resort to the most unscrupulous means, and cited instances in support of his charge that the demands made by the company upon the profession, with respect to patents, etc., were of a character which stamped them as most outrageous impositions.

After referring to the developments attending the investigation now being made by a committee, constituted at the last meeting of the American Association at Saratoga (of which committee he was made a member), Dr. Winder appealed to the feeling of State pride of the profession in Maryland, and urged his hearers to unite with their brethren of other States, so that that power which springs from unity of purpose and concentration of action may make itself felt.

Addresses endorsing the sentiments expressed by Dr. Winder, and in further explanation and advocacy of the protective movement, were made by Dr. A. J. Volck, Dr. E. P. Keech, Dr. M. W. Foster, Dr. R. F. Hunt, and Dr. D. Genese.

### THE REPORT ON HISTOLOGY, ANATOMY, ETC.

Dr. R. Grady, by way of substantiating the statements contained in the report previously read by him, here read quotations from cyclopædias, theological dictionaries, and other sources.

### *Friday, December* 6, 1889.—*Evening Session.*

After the transaction of routine business, the installation of officers, etc., the association and its guests were entertained by W. Xavier Sudduth, A.M., M.D., D.D.S., of Philadelphia, in an essay on life, from a biological point of view, with stereopticon illustrations.

Election of officers of the Maryland State Dental Association for 1890: J. Emory Scott, D.D.S., President; C. B. King, D.D.S., First Vice-President; John C. Uhler, M.D., D.D.S., Second Vice-President; W. W. Dunbracco, D.D.S., Recording Secretary; J. J. Williams, M.D., D.D.S., Corresponding Secretary; F. H. Davy, D.D.S., Treasurer.

*Executive Committee.*—C. M. Gingrich, D.D.S., A. Price, D.D.S., A. P. Gove, D.D.S.

W. W. Dunbracco, *Secretary.*

